# Liver abscess caused by *Klebsiella pneumoniae*: two case reports

**DOI:** 10.1186/1757-1626-2-6879

**Published:** 2009-05-15

**Authors:** Francesco Casella, Luigi Finazzi, Valentina Repetti, Giampaolo Rubin, Maria DiMarco, Tiziana Mauro, Raffaello Furlan

**Affiliations:** Divisione di Medicina Generale, Azienda Ospedaliera Bologninivia Paderno 21-24068 Seriate (Bg)Italy

## Abstract

**Introduction:**

The present case reports highlight the peculiar aspect of *Klebsiella pneumoniae* liver abscess, an emerging disease in United States and Western countries.

**Case presentation:**

We report two cases of Asiatic patients with *Klebsiella*-associated liver abscesses evaluated at our institution over a one-year period. Both of them had non-specific clinical symptoms at presentation, a peculiar ultrasonographic appearance and successful treatment with early percutaneous drainage.

**Conclusion:**

*Klebsiella* related liver abscess is an emerging disease with peculiar clinical features. As compared with other bacterial liver abscesses, *Klebsiella pneumonia* associated pyogenic liver abscess has distinctive risk factors, unique ultrasonographic and computed tomography features and different prognosis.

## Introduction

Pyogenic liver abscess (PLA) is a potentially life-threatening disease that may be caused by bacterial or fungal organisms. The most common bacteria isolated from liver abscess patients are gram-negative rods. Until the end of the last century, *Escherichia coli* was recognized as the predominant cause of bacterial liver abscesses [1], but more recently *Klebsiella pneumoniae* was found to be the leading pathogen in Asia and Western countries [2,3].

Here we describe two cases of *Klebsiella*-associated PLA who were investigated and successfully treated in our department.

## Case presentation

### Case report 1

A 29 year old Indian man reported a 4-day history of fever, chills and fatigue. He was previously in good health. He immigrated to Italy nine years before. He denied any recent foreign travel.

On general examination temperature was 40°C; pulmonary, cardiac and abdominal examinations revealed no abnormality. Laboratory tests were remarkable for a leukocytosis (14040 cells/mm^3^), total bilirubin 1.6 mg/dl, alanine transferase 100 U/L, aspartate transferase 69 U/L. Chest X-ray showed an elevated right hemidiaphragm. The patient was empirically treated with intravenous levofloxacin and ceftriaxone but spiking fever persisted.

**Figure 1.**
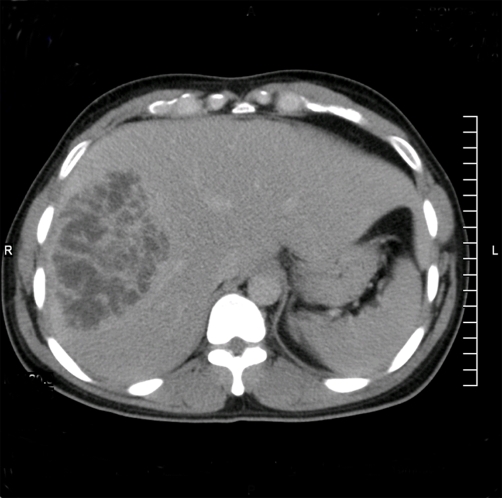
**Computed Tomography scan of a 29 year old man with Klebsiella pneumoniae liver abscess.** Abdominal Computed Tomography scan showing a 10 cm diameter Klebsiella related abscess. Notice, as a distinctive feature, the hypodense lesion with internal septa and irregular margins.

Three days after hospitalization the patient developed right upper quadrant abdominal pain. An abdominal ultrasound (US) revealed a 10 cm diameter lesion in the right lobe of the liver. A Computed Tomography (CT) scan showed a 10 cm hypodense lesion with internal septa (Figure 1). A US-guided percutaneous drainage was placed yielding 150 cc of purulent fluid, but no pathogen was isolated from cultures of aspirated pus. *Entamoeba histolytica* serology was negative. Two out of three blood cultures grew *Klebsiella pneumoniae* resistant to levofloxacin. Therefore, the patient was given intravenous gentamicin and metronidazole.

This therapy led to a clinical improvement and percutaneous drainage was removed. An abdominal US before discharge revealed significant reduction in abscess size. The patient was discharged and antibiotics were switched to oral ciprofloxacin and metronidazole for a 6-week course. A follow-up US after antibiotic therapy showed resolution of the abscess. Patient turned to his usual state of health.

### Case report 2

A 76 year old Chinese man presented with a 5-day history of spiking fever and chills. He was previously in good health; past medical history revealed a cholecystectomy for cholelithiasis ten years before.

General examination showed significant hypotension (85/50 mmHg) and a temperature of 41°C; the remainder of his examination was unremarkable. Laboratory tests revealed elevated C Reactive Protein (31 mg/dl; normal value<1), abnormal serum creatinine (1.8 mg/dl), aspartate transferase (139 U/L), alanine transferase (110 U/L), phosphatase alkaline (823 U/L) and total bilirubin (2.12 mg/dl). The patient was treated with fluids and empiric antibiotic therapy with Piperacillin-Tazobactam was started.

Abdominal US showed a 3 cm diameter lesion in the right lobe of the liver. Abdominal CT scan confirmed the presence of a 3 cm hypodense lesion with irregular margins strongly suspicious for metastatic liver neoplasm. A percutaneous drainage catheter was placed. Diagnostic aspiration of the lesion yielded 50 cc of purulent material that grew *Klebsiella pneumoniae*. Three blood cultures grew *Klebsiella pneumoniae* sensitive to all antibiotics. *Entamoeba histolytica* serology was negative.

Patient's clinical conditions improved; an abdominal US before discharge showed complete resolution of liver abscess. Patient was discharged and treated as an outpatient with levofloxacin and metronidazole for 4 weeks.

## Conclusions

First reported from Taiwan as a solitary organism in PLA [4], in recent studies *Klebsiella pneumoniae* was recognized as the leading pathogen of PLA in Asia and Western countries [2,3]. Furthermore, it was found to be associated with Asian ethnicity, diabetes mellitus and cryptogenic liver abscesses [2,3,5]. Conversely, the presence of biliary disease and coexisting malignancy was recognized as a predictive parameter of *Escherichia coli* PLA [3].

Patients often complain of vague constitutional symptoms, such as fever and fatigue, while only one-half of cases presents with more specific clinical clues as right upper quadrant pain, jaundice and hepatomegaly [2,6]. Only non-specific clinical symptoms were identified in our cases, even in the first patient who had such a large PLA (10 cm). For this reason the absence of right upper abdominal quadrant findings does not exclude a liver abscess. Moreover a PLA should always be considered in the differential diagnosis of a fever of unknown origin.

Distinctive US and CT features have been reported in *Klebsiella pneumoniae* PLA [7,8]. A predominantly solid US appearance of the lesion with irregular margins was observed in *Klebsiella*-associated liver abscesses, possibly due to their failure of liquefaction [7]. As compared with other bacterial abscesses, a smaller quantity of pus obtained at initial aspiration was reported in *Klebsiella* PLA [7], which can be related to their predominant solid consistency made up of aggregation of multiple locules that do not communicate. These features were observed in our patients, particularly in the second one where a liver neoplasm was considered in the differential diagnosis.

Some studies showed a better prognosis for patients with *Klebsiella*-associated PLA than for other bacterial liver abscesses [4,9]. However, a higher incidence of metastatic infections at other sites was shown in patients with *Klebsiella* related PLA [9]. The most common manifestations of metastatic infection are endophthalmitis, meningitis and brain abscess [10]. Major virulence factors of *Klebsiella pneumoniae* were shown to be associated with a metastatic disease. Capsular K1 serotype is a significant virulence determinant for the development of septic ocular and central nervous system complications from PLA [10]. Furthermore a number of clinical studies pointed to a relationship between the hypermucoviscosity phenotype of *Klebsiella pneumoniae* and the presence of an invasive disease with metastatic infection [11]. *Klebsiella pneumoniae* strains possess the hypermucosviscosity phenotype if they are capable of producing a mucoviscous exopolysaccharide web. In the microbiology laboratory, these strains grow in sticky colonies on agar plates and are identified by a string test. Although diabetes mellitus was recognized as a major risk factor for *Klebsiella* related PLA, the data are conflicting as to whether it is associated with an invasive and metastatic infection [10]. None of our patients had diabetes mellitus, none of them experienced ocular or neurologic complications.

A significant reduction of mortality has occurred for all pyogenic liver abscesses since 1950, possibly related to the advent of percutaneous drainage and broad spectrum antibiotics [12]. In contrast with amoebic abscess, drainage of pyogenic abscess is essential in most cases. This issue is emphasized by Cheng et al. (2003) [13] who observed that early drainage can significantly improve prognosis. Percutaneous drainage is a relatively low-risk and effective method. It has gradually replaced surgical procedures since it was shown to reduce costs and length of hospital stay [14]. Surgical drainage should be considered in patients with multiple and large liver abscesses and when there is no response to percutaneous drainage [15]. In addition to drainage, treatment of *Klebsiella* liver abscess requires parenteral antibiotics. Since community-acquired *Klebsiella* strains are resistant to first generation cephalosporin and ampicillin but rarely produce extended-spectrum beta-lactamases (ESBL), a combination of an extended spectrum beta-lactam and an aminoglycoside is the preferred regimen. Although aminoglycosides penetrate the abscess cavity poorly, they can rapidly eradicate organisms in the bloodstream and decrease the risk of metastatic infections.

## List of abbreviations

CT: Computed Tomography
ESBL: extended-spectrum beta-lactamases
PLA: Pyogenic liver abscess
US: Ultrasound
